# Supplementary material for: Corneal Adhesion Possesses the Characteristics of Solid and Membrane
Source: Bioengineering (Basel). 2022 Aug 16;9(8):394. doi: 10.3390/bioengineering9080394 (PMC9405176; doi:10.3390/bioengineering9080394)
Supplement: Supplementary file 1 [file bioengineering-09-00394-s001.zip › bioengineering-1843201-supplementary.pdf]

bioengineering

Supplementary Information for: Corneal adhesion possesses the characteristics of solid and membrane

Supplementary information I: Measuring the adhesion behavior of cornea performed the indentation-detachment test

This section was the supplementary for the sketching picture (Figure 1), which was used to describe the pull-off test performed to determine the adhesion behavior of cornea. Each eye specimen, in this study, was tested by seven different punches with different radii, 0.5 mm, 1.0 mm, 2.0 mm, 2.5 mm, 3.0 mm, 4.0 mm, and 5.0 mm, respectively. The related images captured through CCD were as shown in Figure S1.

All seven punches were used to determine the corneal adhesion behavior during the eye was under the normal IOP of 20 mmHg and 30 mmHg. Refer to the related literature, the normal IOP varies from individual to individual and is in the range of 1.5 – 4 kPa, i.e.., approximately 10 – 30 mmHg[27]. In order to verify the influence from the variation of IOP, four punches, respectively with the radii of 1.0 mm, 2.0 mm, 3.0 mm, and 4.0 mm, were also used to performed the pull-off test during the eye was under the higher ***P*** values of 40 mmHg, 50 mmHg, and 60 mmHg.

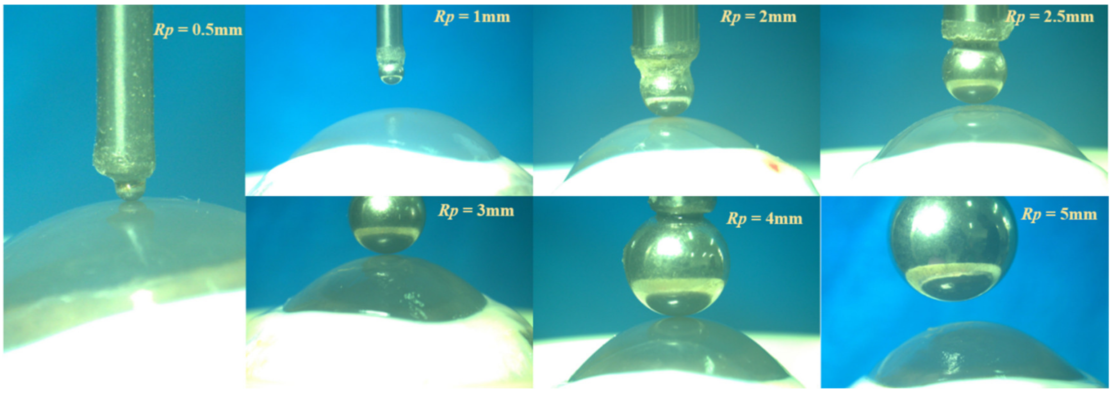

Figure S1. Images captured through CCD. The seven pictures show the comparison of the sizes between the punches and the cornea.

Supplementary information II: Appendix of the mathematical derivation for the adhesion force between the interface

As shown in Figure S2, a flat-cylindric punch indents a semi-infinite solid, the related contact force ***F***, solved by Sneddon (1965) [23], can be written as

$$F = \frac{4G_m a\delta}{1-\upsilon_m}, \tag{S1}$$

where ***a*** and ***δ*** are the contact radius and indentation depth, respectively. And $\boldsymbol{G_m}$ and $\boldsymbol{\upsilon_m}$ are the shear modulus and Poisson's ratio of the material, respectively. The shear modulus $\boldsymbol{G_m}$ can be described by the elastic modulus ***E*** as $\boldsymbol{G_m} = \boldsymbol{E_m} / 2(1 + \boldsymbol{\upsilon_m})$, thus the contaction ***F*** can also be formulated as

$$F = \frac{2a\delta E_m}{1-\upsilon_m^2}. \tag{S2}$$

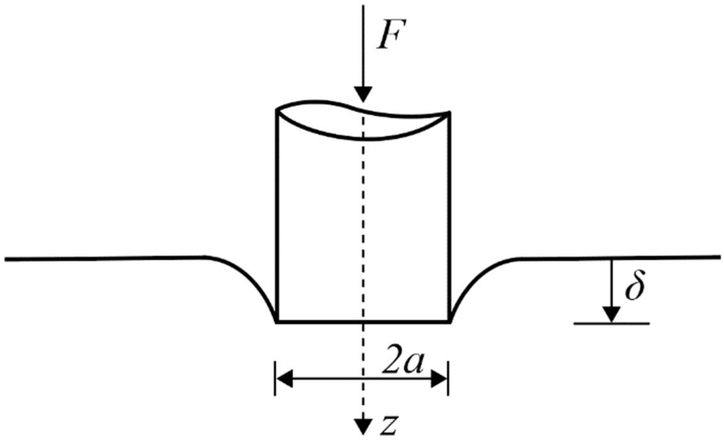

Figure S2. Sketching for a flat-cylindrical punch indents a semi-infinite solid.

According to the elastic theory, the effective elastic modulus $\boldsymbol{E^*}$ of the indentation behavior can be written as

$$\frac{1}{E^*} = \frac{1-\upsilon_p^2}{E_p} + \frac{1-\upsilon_m^2}{E_m}, \tag{S3}$$

where $\boldsymbol{E_p}$ and $\boldsymbol{\upsilon_p}$ are the elastic modulus and Poisson's ratio of the punch, respectively. In case of that the punch is much stiffer than the material, for example, a steel punch with $\boldsymbol{E_p}$ of hundreds of GPa and a biological soft tissue with $\boldsymbol{E_m}$ of hundreds of kPa, the first term of the right hand in *Eq*. (S3) can be neglected. In this sense, the effective $\boldsymbol{E^*}$ can also be simply formulated as $\boldsymbol{E^*} \approx \boldsymbol{E_m} / (1-\boldsymbol{\upsilon^2_m})$. Thus, *Eq*. (S2) is simply written as

$$F = 2a\delta E^* . \quad \text{(S4)}$$

If the mechanical property of the material appears linear behavior, the related stiffness $\boldsymbol{K}$ can be defined as

$$K = \frac{F}{\delta} = 2aE^* . \quad \text{(S5)}$$

Kendall (1971) studied the adhesion behavior between a rigid flat-cylindric punch and an elastic substrate, through observing the punch contacted to and then detached from the substrate [27]. Based on the Griffith's energy criterion, he found that the pull-off force (i.e., adhesion force $\boldsymbol{F_{ad}}$) of the punch detached from the substrate is

$$F_{pull-off} = F_{ad} = -\sqrt{8\pi\gamma E^* a^3} , \quad \text{(S6)}$$

where γ is the work of adhesion, which means that the adhesion energy per unit contaction area. The minus sign means the tensile direction. The corresponding deriving process is as follows.

A rigid punch indents to a soft material and then detaches from it, as shown in Figure S3, the related total potential energy U_T required for the detachment process is

$$U_T = U_E + U_{Surf} + U_F , \quad \text{(S7)}$$

where U_E, U_{Surf}, and U_F are the elastic strain energy of the substrate, the additional surface energy, and the work of external force, respectively.

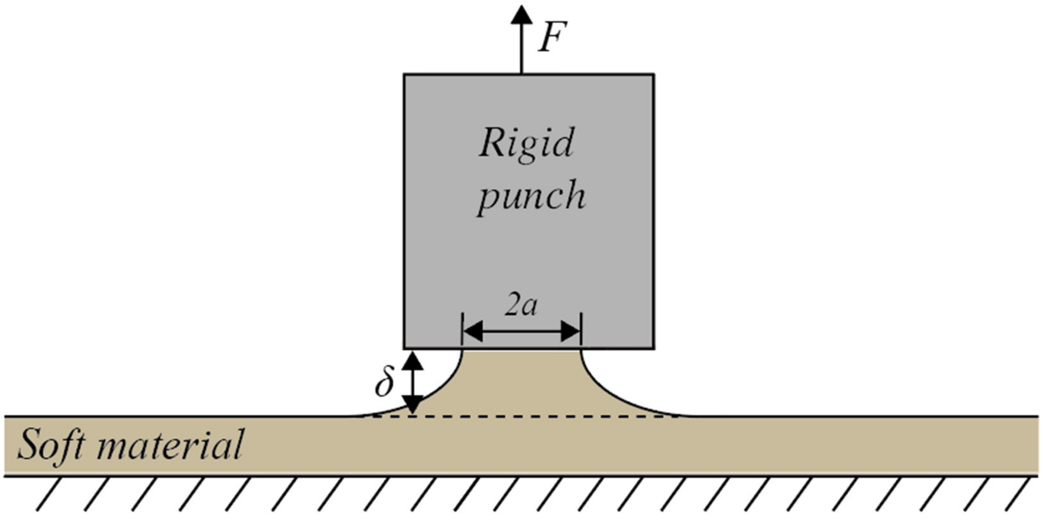

Figure S3. Sketching for a rigid punch detaches from a soft material.

For the linear adhesion behavior, U_E, U_F, and U_{Surf} are respectively formulated as

$$\begin{cases} U_E = \frac{1}{2}F\delta = \frac{1}{2}\frac{F^2}{K} \\ U_F = -F\delta = -\frac{F^2}{K} \\ U_{Surf} = \pi a^2 \gamma \end{cases} . \tag{S8}$$

During the detachment, the adhesion force will appear at the critical contact area where the pull-in process transforms into pull-off process and the pull-off force reaches maximum value (Figure 1b). In mathematics, therefore, there is

$$\begin{aligned} \frac{\partial U_T}{\partial a} = 0 \Rightarrow \frac{\partial}{\partial a}\left(\frac{F^2}{2K} - \frac{F^2}{K} + \pi a^2 \gamma\right) \\ = \frac{\partial}{\partial a}\left(\pi a^2 \gamma - \frac{F^2}{4aE^*}\right) \\ = 2\pi\gamma a + \frac{F^2}{4E^*}\frac{1}{a^2} = 0 \\ \Rightarrow F = -\sqrt{8\pi\gamma E^* a^3} \end{aligned} . \tag{S9}$$

Supplementary information III: Appendix derivation related to the contact behavior of a rigid sphere indents a soft spherical solid (JKR model)

As shown in Figure S4, Hertz (1896) [22] first solved the contaction problem between two rigid spheres, which can be written as

$$\begin{cases} a^3 = \dfrac{3FR}{4E^*} \\ \delta = \dfrac{a^2}{R} \end{cases}, \tag{S10}$$

where $\boldsymbol{R}$ is the derived radius, $1/\boldsymbol{R} = 1/\boldsymbol{R_p} + 1/\boldsymbol{R_m}$. Based on the Hertz solution, Johnson et al. (1971) [6] reported the solution of the contaction between a rigid sphere and a compliance solid, i.e., the Johnson-Kendall-Roberts (JKR) model formulated as

$$\begin{cases} a^3 = \dfrac{3R}{4E^*}\left[F + 3\gamma\pi R + \sqrt{6\gamma\pi RF + (3\gamma\pi R)^2}\right] \\ \delta = \dfrac{a^2}{R} - \sqrt{\dfrac{2\pi\gamma a}{E^*}} \end{cases}. \tag{S11}$$

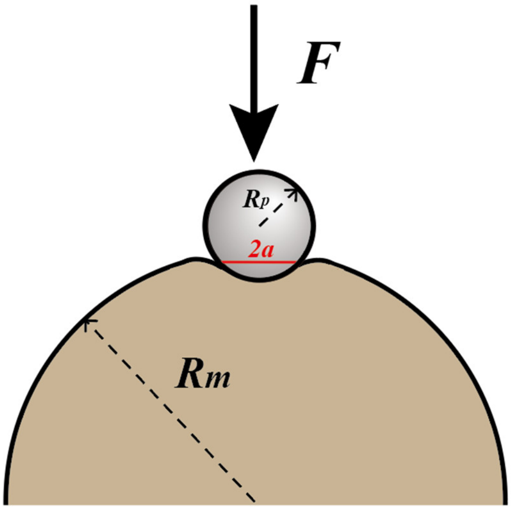

Figure S4. Sketching for the contaction mechanics between two spheres**.**

In JKR model, when $\gamma = 0$ the formula reverts to the simple Hertz equation. At zero applied load, the contact area is finite and given by

$$a_0^3 = \frac{3R}{4E^*}(6\gamma\pi R), \tag{S12}$$

where $\boldsymbol{a_0}$ is contact radius when applied force is zero. In this study, *Eq*. (S12) is used to calculate the effective elastic modulus $\boldsymbol{E^*}$ of cornea.

During the punch detaches from the compliance soft solid, the applied load is made negative and the contact radius decreases. The related limiting condition of JKR model is as follows

$$\sqrt{6\gamma\pi RF+\left(3\gamma\pi R\right)^{2}}\geq 0\Rightarrow F\geq -\frac{3}{2}\gamma\pi R. \tag{S13}$$

Separation will just occur when the pull-off force or adhesion force reaches the maximum value in *Eq*. (S13), i.e.,

$$F_{pull-off}=F_{ad}=-\frac{3}{2}\gamma\pi R. \tag{S14}$$

Another method can also obtain the adhesion value of JKR model is $1.5\gamma\pi R$ through analyzing the equilibrium of the detachment process. As shown in Figure S4 and Figure 1c, a rigid sphere indents and then detaches from a soft spherical solid, the sum force is contributed from two terms, the Hertz contact force, and the interface adhesion force. Accordingly, the related sum force is formulated as

$$F^{\text{solid}}=\underbrace{\frac{4E^{*}a^{3}}{3R}}_{Hertz}-\underbrace{\sqrt{8\gamma\pi E^{*}a^{3}}}_{Kendall}. \tag{S15}$$

The separation happens when *Eq*. (S15) appears extreme value, that means

$$\begin{aligned}&\frac{\partial F}{\partial a}=\frac{4E^{*}}{R}a^{2}-3\sqrt{2\pi\gamma E^{*}}\sqrt{a}=0\\&\Rightarrow a_{c}=\left(\frac{9}{8}\frac{\gamma\pi R^{2}}{E^{*}}\right)^{\frac{1}{3}}\end{aligned}, \tag{S16}$$

where $\boldsymbol{a_c}$ is the critical contact radius. Substituting the formula *Eq*. (S16) of a_c into the equilibrium equation *Eq*. (S15), the adhesion force is solved as follows

$$\begin{aligned}F_{ad}=F_{\max}^{\text{solid}}&=\frac{4E^{*}a_{c}^{3}}{3R}-\sqrt{8\gamma\pi E^{*}a_{c}^{3}}\\&=\frac{4E^{*}}{3R}\frac{9}{8}\frac{\gamma\pi R^{2}}{E^{*}}-\sqrt{8\gamma\pi E^{*}\frac{9}{8}\frac{\gamma\pi R^{2}}{E^{*}}}\\&=\frac{3}{2}\gamma\pi R-3\gamma\pi R\\&=-\frac{3}{2}\gamma\pi R\end{aligned}. \tag{S17}$$

Supplementary Information IV: **The comparisons of the corneal adhesion forces between the experimental and the theoretical data**

This section is the supplementary for the comparison results shown as in Figure 4. In this study, we calculated the corneal adhesion force $\boldsymbol{F_{ad}}$ through the JKR and the modified-JKR models. The related JKR solutions were obtained through the classical formula of $1.5\boldsymbol{\gamma\pi R}$, while the related modified-JKR solutions were obtained through the formula of *Eq*. (2) proposed here with the constant coefficient $\boldsymbol{\alpha}$ were equal to 0.1, 0.2, 0.3, 0.4, and 0.5. The detailed calculation results were as shown in Figure S5.

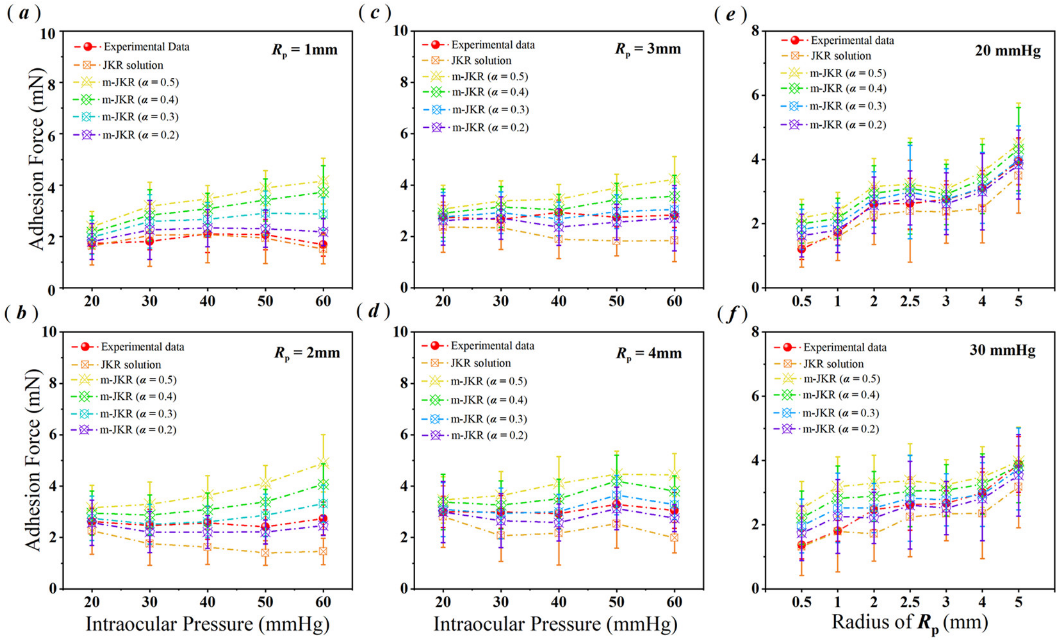

Figure S5. Supplementary comparisons of the corneal adhesion force $\boldsymbol{F_{ad}}$ obtained from the experiment and theories, including the JKR solutions and the modified JKR solutions with $\boldsymbol{\alpha}$ equals to 0.1, 0.2, 0.3, 0.4, and 0.5. (**a**)-(**d**) show the comparisons with different IOPs. (**e**)-(**f**) show the comparisons of the scale effect of the corneal adhesion under the normal IOP of 20 mmHg and 30 mmHg (the data presented as **mean** ± **s.d.**, and the abbreviations of JKR sol and m-JKR indicate the solutions from JKR and modified-JKR models, respectively).

In terms of the modified-JKR model used here, the coefficient $\boldsymbol{\alpha}$ indicated the degree of the corneal adhesion trending to the characteristic of membrane. The classical JKR model, $1.5\boldsymbol{\gamma\pi R}$, could be also obtained by the modified-JKR model with $\boldsymbol{\alpha} = 0$. Figure 4 and Figure S5 in this study, therefore, showed the comparisons between the experimental data and the modified-JKR solutions with $\boldsymbol{\alpha}$ were in the range from 0 to 0.5.

Supplementary Information V: The detailed derivation process of the surface tension term in *Eq.* 1

In the study, the modified JKR model was derived from the force analysis of a pure membranous material. Imaging the situation that a spherical rigid punch contacted with a spherical thin-film, which was inflated by air, such as, an inflated balloon. The equilibrium of the spherical thin-film was affected by the air pressures, the indentation loading (Hertz force), the surface adhesion (Kendall adhesion), and the membranous tension (Figure S6).

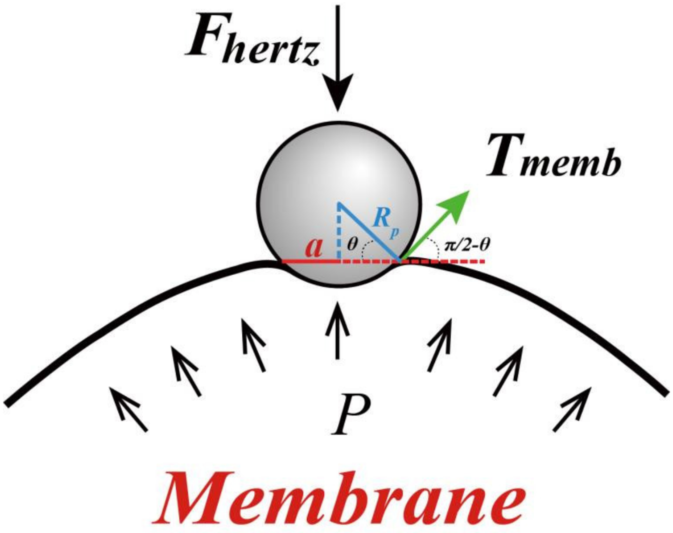

Figure S6. The contaction between a small rigid sphere between an inflated thin-spherical film.

According to the sketching of the above force analysis, and assumption the indentation direction was the positive, the related equilibrium equation could be written as

$$F=\frac{4E^{*}a^{3}}{3R}-\sqrt{8\pi\gamma E^{*}a^{3}}-P\pi a^{2}-2\pi a\left(\frac{PR_{m}}{2}\right)\sin\left(\frac{\pi}{2}-\theta\right) \quad \text{(S18)}$$

where a is the contaction radius, γ the work of adhesion, P the air pressure, E^{*} and R the effective elastic modulus and the radius, respectively. Here, $1/E^{*} = (1-\upsilon^{2}_{p})/E_{p} + (1-\upsilon^{2}_{m})/E_{m}$ and $1/R = 1/R_{p} + 1/R_{m}$. In the *Eq*. (S18), the first term was the classical Hertz (1896) [22] contaction force, the section term was the classical interfacial adhesion derived by Kendall (1971) [27], the third term was the air pressure applied on the contaction region, and the fourth term was the membranous tension. The third and the fourth terms were correlated with the characteristics of the membrane.

According to the Fung (2013) [24], the tension of the per unit length, related to the uniform material, was formulated as $PR_{m}/2$. The outer perimeter contaction region, as shown in Fig. S6, was 2πa. Therefore, the membranous tension applied the whole contaction outer circle was

$2\pi a(PR_m/2)$. The fourth term was the membranous tension projected to the indentation direction. According to the geometry as shown in Fig. S6, one could easily obtain that

$$\sin\left(\frac{\pi}{2}-\theta\right)=\cos\theta=\frac{a}{R_p} \quad \text{(S19)}$$

Substituting *Eq*. (S19) into *Eq*. (S18), we obtained

$$\begin{aligned} F&=\frac{4E^*a^3}{3R}-\sqrt{8\pi\gamma E^*a^3}-P\pi a^2-2\pi a\left(\frac{PR_m}{2}\right)\sin\left(\frac{\pi}{2}-\theta\right) \\ &=\frac{4E^*a^3}{3R}-\sqrt{8\pi\gamma E^*a^3}-P\pi a^2-2\pi a\left(\frac{PR_m}{2}\right)\frac{a}{R_p} \\ &=\frac{4E^*a^3}{3R}-\sqrt{8\pi\gamma E^*a^3}-P\pi a^2\left(1+\frac{R_m}{R_p}\right) \\ &=\frac{4E^*a^3}{3R}-\sqrt{8\pi\gamma E^*a^3}-P\pi a^2\left(\frac{R_p+R_m}{R_p}\right) \end{aligned} \quad \text{(S20)}$$

The effective radius could be rewritten as

$$\frac{1}{R_p}+\frac{1}{R_m}=\frac{R_p+R_m}{R_pR_m}=\frac{1}{R} \quad \text{(S21)}$$

Thus, we have

$$\frac{R_p+R_m}{R_p}=\frac{R_m}{R} \quad \text{(S22)}$$

Substituting *Eq*. (S22) into *Eq*. (S21), we obtained the simplification formula

$$F=\frac{4E^*a^3}{3R}-\sqrt{8\pi\gamma E^*a^3}-P\pi a^2\frac{R_m}{R} \quad \text{(S23)}$$

The *Eq*. (S23) could be used to describe the contaction problem between a small rigid sphere and a soft spherical pure-membrane inflated by air pressures. Physiologically, the intact cornea was inflated by intraocular pressures (IOPs), which was similar to the inflation situation of a balloon. Different from a pure membrane, the corneal thickness should not be ignored. Accordingly, we introduced a power-coefficient $\boldsymbol{\alpha}$ into *Eq*. (S23) to estimate the tendency degree of the cornea trended to the membrane. Therefore, replacing the Rm term by the corneal radius of curvature R_c in *Eq*. (S23), we obtained the equilibrium formula of an intact inflated cornea contacted by a small rigid sphere, i.e., *Eq*. (1) in the manuscript.

Supplementary Information VI: The detailed experimental data related to each sample under 20 mmHg-IOP

Table S1. The detailed experimental data of each cornea under normal IOP of 20 mmHg.

sample	R_p (mm)	δ_m (mm)	R_c (mm)	R (mm)	a_c (mm)	F_{ad} (mN)	γ (mN/mm)	E^* (kPa)
Eye 1-3	0.5	0.031	8.3995	0.4719	0.096	0.972	0.5137	981.73
Eye 3-1	0.5	0.031	9.9042	0.476	0.092	0.2313	0.134	245.82
Eye 3-3	0.5	0.035	9.9042	0.476	0.1131	1.3172	0.5769	995.31
Eye 5-1	0.5	0.04	9.5986	0.4752	0.1008	0.7585	0.4752	867.26
Eye 5-2	0.5	0.029	9.5986	0.4752	0.2619	0.7173	0.0481	64.41
Eye 5-3	0.5	0.019	9.5986	0.4752	0.1026	0.9265	0.2662	367.81
Eye 6-1	0.5	0.03	8.275	0.4715	0.106	1.2544	0.526	489.79
Eye 6-2	0.5	0.029	8.275	0.4715	0.099	1.1709	0.5552	572.39
Eye 6-3	0.5	0.029	8.275	0.4715	0.092	1.1946	0.6492	676.25
Eye 7-1	0.5	0.022	8.8092	0.4731	0.1115	1.0934	0.3136	303.02
Eye 7-2	0.5	0.024	8.8092	0.4731	0.1465	1.2831	0.2322	152.22
Eye 7-3	0.5	0.026	8.8092	0.4731	0.0985	1.1194	0.4738	529.22
Eye 8-1	0.5	0.032	8.2887	0.4716	0.1365	1.3097	0.3546	302.29
Eye 8-2	0.5	0.032	8.2887	0.4716	0.1035	1.2961	0.6143	589.68
Eye 8-3	0.5	0.037	8.2887	0.4716	0.0915	1.3207	0.9164	804.32
Eye 9-1	0.5	0.03	9.1623	0.4741	0.1175	1.4817	0.5124	369.19
Eye 9-2	0.5	0.031	9.1623	0.4741	0.093	1.5717	0.9024	741.02
Eye 9-3	0.5	0.029	9.1623	0.4741	0.099	1.5404	0.7154	723.08
Eye 10-1	0.5	0.03	9.7264	0.4756	0.081	1.0726	0.7806	732.08
Eye 10-2	0.5	0.03	9.7264	0.4756	0.1015	1.478	0.6941	901.98
Eye 10-3	0.5	0.038	9.7264	0.4756	0.097	1.3224	0.8478	803.09
Eye 1-1	1	0.0372	9.0084	0.9001	0.1534	1.5619	0.3931	854.65
Eye 1-2	1	0.0450	9.0084	0.9001	0.1539	1.2186	0.3686	711.95
Eye 3-2	1	0.0429	11.0478	0.9170	0.1864	2.1772	0.4280	305.92
Eye 4-2	1	0.0320	10.2979	0.9115	0.1440	0.4872	0.1197	226.53
Eye 4-3	1	0.0594	10.2979	0.9115	0.1713	1.2827	0.4135	609.36
Eye 7-1	1	0.0281	9.7906	0.9073	0.1460	1.5094	0.3167	427.81
Eye 7-2	1	0.0264	9.7906	0.9073	0.1420	1.8934	0.3946	951.30
Eye 8-1	1	0.0273	9.6244	0.9059	0.1995	1.6367	0.1787	148.09
Eye 8-2	1	0.0252	9.6244	0.9059	0.1780	1.5161	0.1919	210.53
Eye 8-3	1	0.0294	9.6244	0.9059	0.1815	1.7520	0.2489	262.10

Eye 9-2	1	0.0289	8.5972	0.8958	0.1900	1.6511	0.2104	230.36
Eye 9-3	1	0.0290	8.5972	0.8958	0.2245	1.7461	0.1599	121.11
Eye 10-1	1	0.0300	10.9740	0.9165	0.2030	1.9861	0.2301	282.76
Eye 10-2	1	0.0329	10.9740	0.9165	0.1720	1.9977	0.3536	480.29
Eye 11-1	1	0.0313	8.2727	0.8922	0.1970	1.5566	0.1998	232.66
Eye 11-2	1	0.0292	8.2727	0.8922	0.1865	1.5611	0.2086	295.59
Eye 11-3	1	0.0305	8.2727	0.8922	0.1690	1.7316	0.2943	535.96
Eye 12-1	1	0.0302	9.7043	0.9066	0.1530	2.0842	0.4280	598.80
Eye 12-2	1	0.0298	9.7043	0.9066	0.1685	2.2384	0.3739	659.09
Eye 12-3	1	0.0340	9.7043	0.9066	0.1585	2.1607	0.4654	854.07
Eye 3-1	2	0.0302	10.4245	1.6781	0.2234	2.8601	0.2755	641.43
Eye 4-1	2	0.0273	9.0958	1.6395	0.2175	2.3156	0.2127	326.29
Eye 4-2	2	0.0350	9.0958	1.6395	0.2525	3.1604	0.2761	330.96
Eye 4-3	2	0.0312	9.0958	1.6395	0.2115	2.6650	0.2958	384.76
Eye 5-1	2	0.0387	9.2409	1.6442	0.1960	3.2341	0.5185	671.65
Eye 5-2	2	0.0352	9.2409	1.6442	0.2215	3.2685	0.3732	870.38
Eye 5-3	2	0.0371	9.2409	1.6442	0.2580	3.5919	0.3186	334.25
Eye 6-1	2	0.0327	9.0778	1.6389	0.2700	1.9627	0.1401	196.08
Eye 6-2	2	0.0425	9.0778	1.6389	0.2730	1.6400	0.1488	244.16
Eye 6-3	2	0.0281	9.0778	1.6389	0.1335	2.2115	0.5550	877.58
Eye 7-1	2	0.0269	8.6938	1.6260	0.1990	2.2464	0.2429	271.87
Eye 7-2	2	0.0267	8.6938	1.6260	0.2190	1.7821	0.1579	328.13
Eye 7-3	2	0.0316	8.6938	1.6260	0.2235	2.2932	0.2309	374.74
Eye 8-1	2	0.0267	9.3296	1.6469	0.2180	2.8616	0.2559	350.97
Eye 8-2	2	0.0325	9.3296	1.6469	0.2605	2.9431	0.2243	294.41
Eye 8-3	2	0.0316	9.3296	1.6469	0.2450	2.4084	0.2018	227.50
Eye 9-1	2	0.0489	9.8072	1.6612	0.2370	2.4542	0.3401	408.69
Eye 9-2	2	0.0381	9.8072	1.6612	0.2745	3.1586	0.2542	365.46
Eye 9-3	2	0.0396	9.8072	1.6612	0.2670	2.8651	0.2533	303.00
Eye 1-2	2.5	0.0525	10.0694	2.0028	0.1853	3.2792	0.1995	32.48
Eye 1-3	2.5	0.0490	10.0694	2.0028	0.1836	4.5601	0.2639	101.64
Eye 2-2	2.5	0.0271	9.7732	1.9908	0.1990	1.8844	0.2052	164.19
Eye 3-1	2.5	0.0371	9.7479	1.9897	0.1935	1.8030	0.2843	367.56
Eye 5-2	2.5	0.0364	9.5760	1.9824	0.1923	1.9253	0.3018	665.70
Eye 6-1	2.5	0.0382	9.3548	1.9728	0.1800	1.9408	0.3642	599.83
Eye 7-1	2.5	0.0423	9.4994	1.9791	0.1485	1.8533	0.5658	296.51

Eye 7-2	2.5	0.0323	9.4994	1.9791	0.2158	2.3856	0.2635	343.37
Eye 8-1	2.5	0.0331	9.4943	1.9789	0.1835	2.4589	0.3847	585.18
Eye 8-2	2.5	0.0319	9.4943	1.9789	0.1810	2.5905	0.4015	548.53
Eye 10-1	2.5	0.0282	9.5441	1.9811	0.1755	2.2392	0.3263	612.70
Eye 10-2	2.5	0.0292	9.5441	1.9811	0.1750	2.2745	0.3452	352.93
Eye 11-3	2.5	0.0448	9.7273	1.9889	0.2460	4.1246	0.4860	721.12
Eye 12-1	2.5	0.0295	9.6605	1.9860	0.1900	2.4756	0.3220	486.95
Eye 12-2	2.5	0.0350	9.6605	1.9860	0.2650	2.7834	0.2208	239.61
Eye 13-3	2.5	0.0360	9.8607	1.9944	0.2040	2.2522	0.3101	503.25
Eye 1-1	3	0.0368	9.6991	2.2913	0.2600	2.8191	0.2443	326.49
Eye 1-2	3	0.0294	9.6991	2.2913	0.2955	2.4336	0.1304	228.67
Eye 1-3	3	0.0305	9.6991	2.2913	0.3035	2.5075	0.1321	173.21
Eye 2-1	3	0.0350	9.3077	2.2687	0.2800	2.3928	0.1700	243.25
Eye 2-2	3	0.0334	9.3077	2.2687	0.2730	1.9648	0.1401	215.86
Eye 3-1	3	0.0327	9.5402	2.2823	0.3025	1.9777	0.1125	145.71
Eye 3-2	3	0.0296	9.5402	2.2823	0.2945	2.4152	0.1312	272.52
Eye 4-1	3	0.0331	8.5696	2.2221	0.2745	2.5212	0.1763	427.34
Eye 4-2	3	0.0302	8.5696	2.2221	0.3055	2.4319	0.1252	199.60
Eye 5-1	3	0.0308	9.7508	2.2942	0.2550	2.5703	0.1938	495.85
Eye 5-2	3	0.0319	9.7508	2.2942	0.2770	2.8178	0.1865	340.80
Eye 5-3	3	0.0329	9.7508	2.2942	0.2955	2.7933	0.1675	318.50
Eye 6-1	3	0.0362	9.3855	2.2733	0.2680	3.7598	0.3016	599.43
Eye 6-2	3	0.0339	9.3855	2.2733	0.2970	2.8141	0.1721	346.78
Eye 7-1	3	0.0296	9.7838	2.2960	0.2715	2.8995	0.1853	345.24
Eye 7-2	3	0.0327	9.7838	2.2960	0.3120	2.5379	0.1357	271.39
Eye 7-3	3	0.0423	9.7838	2.2960	0.2445	3.1165	0.3510	787.10
Eye 8-1	3	0.0516	9.9080	2.3028	0.2845	2.3521	0.2387	480.15
Eye 8-2	3	0.0546	9.9080	2.3028	0.2410	2.6310	0.3937	656.81
Eye 8-3	3	0.0495	9.9080	2.3028	0.2585	2.8922	0.3410	689.13
Eye 9-2	3	0.0409	9.3521	2.2714	0.2565	2.9733	0.2942	676.75
Eye 9-3	3	0.0368	9.3521	2.2714	0.2565	3.1253	0.2782	518.57
Eye 10-1	3	0.0375	9.3353	2.2704	0.2535	2.4300	0.2257	397.04
Eye 10-2	3	0.0335	9.3353	2.2704	0.3120	3.6115	0.1978	323.56
Eye 10-3	3	0.0371	9.3353	2.2704	0.2275	3.5816	0.4086	590.24
Eye 11-1	3	0.0300	9.1342	2.2583	0.2850	2.5047	0.1472	325.48
Eye 11-2	3	0.0321	9.1342	2.2583	0.2940	2.5733	0.1521	242.03

Eye 1-1	4	0.0475	9.4516	2.8105	0.3307	3.1079	0.2149	258.99
Eye 1-2	4	0.0357	9.4516	2.8105	0.3176	2.2022	0.1241	135.45
Eye 2-1	4	0.0571	9.6066	2.8241	0.3201	3.7875	0.3360	370.14
Eye 2-2	4	0.0414	9.6066	2.8241	0.3139	3.0860	0.2063	349.58
Eye 2-3	4	0.0697	9.6066	2.8241	0.3951	3.4197	0.2430	221.54
Eye 7-1	4	0.0348	9.8056	2.8411	0.3522	3.4320	0.1533	217.31
Eye 7-3	4	0.0363	9.8056	2.8411	0.3558	3.2206	0.1470	187.42
Eye 9-1	4	0.0382	9.9393	2.8522	0.2993	3.8109	0.2586	574.97
Eye 9-2	4	0.0360	9.9393	2.8522	0.3685	3.1514	0.1330	141.05
Eye 9-3	4	0.0350	9.9393	2.8522	0.3053	2.8353	0.1695	472.66
Eye 9-1	4	0.0348	9.2548	2.7929	0.2500	2.0359	0.1804	466.02
Eye 9-2	4	0.0419	9.2548	2.7929	0.3045	2.6782	0.1926	470.78
Eye 9-3	4	0.0415	9.2548	2.7929	0.2375	2.4930	0.2919	610.43
Eye 10-2	4	0.0454	9.7173	2.8336	0.3050	4.1876	0.3253	615.29
Eye 10-3	4	0.0447	9.7173	2.8336	0.3635	3.8141	0.2054	463.95
Eye 11-1	4	0.0404	9.3238	2.7991	0.3440	2.7567	0.1498	284.05
Eye 11-2	4	0.0385	9.3238	2.7991	0.3320	2.7852	0.1548	384.97
Eye 11-3	4	0.0391	9.3238	2.7991	0.2445	2.7998	0.2915	563.51
Eye 12-2	4	0.0735	9.2800	2.7952	0.2980	3.0443	0.4010	664.31
Eye 12-3	4	0.0579	9.2800	2.7952	0.3340	3.3706	0.2784	587.87
Eye 13-1	4	0.0429	9.0554	2.7745	0.2385	2.5008	0.3002	468.80
Eye 13-2	4	0.0350	9.0554	2.7745	0.3135	2.9726	0.1685	294.13
Eye 1-1	5	0.0369	8.6917	3.1741	0.2990	4.0790	0.2680	498.91
Eye 1-2	5	0.0369	8.6917	3.1741	0.3005	3.8632	0.2513	489.97
Eye 1-3	5	0.0381	8.6917	3.1741	0.3540	3.9562	0.1914	214.24
Eye 2-1	5	0.0487	9.8306	3.3143	0.3735	5.0833	0.2824	330.63
Eye 2-2	5	0.0447	9.8306	3.3143	0.4160	4.4950	0.1848	193.29
Eye 2-3	5	0.0423	9.8306	3.3143	0.4375	5.1808	0.1822	214.58
Eye 3-1	5	0.0310	9.7860	3.3092	0.3060	2.6110	0.1376	370.34
Eye 3-2	5	0.0388	9.7860	3.3092	0.4285	2.6002	0.0875	108.31
Eye 3-3	5	0.0385	9.7860	3.3092	0.3420	3.6812	0.1929	236.71
Eye 4-1	5	0.0373	10.1445	3.3492	0.3430	2.8982	0.1462	233.66
Eye 4-2	5	0.0358	10.1445	3.3492	0.3230	3.0751	0.1679	331.49
Eye 4-3	5	0.0345	10.1445	3.3492	0.3080	3.4237	0.1982	375.28
Eye 4-4	5	0.0339	10.1445	3.3492	0.3280	3.4342	0.1722	462.20
Eye 5-2	5	0.0331	9.9543	3.3282	0.3380	2.9234	0.1348	395.55

Eye 5-4	5	0.0346	9.9543	3.3282	0.2605	4.0008	0.3247	615.54
Eye 6-1	5	0.0585	8.8753	3.1982	0.3575	3.4653	0.2525	276.82
Eye 6-2	5	0.0442	8.8753	3.1982	0.3205	3.9452	0.2702	466.56
Eye 6-3	5	0.0392	8.8753	3.1982	0.2980	3.7206	0.2614	525.04
Eye 6-4	5	0.0475	8.8753	3.1982	0.4310	4.0401	0.1644	152.26
Eye 7-1	5	0.0658	8.6379	3.1669	0.2875	4.1067	0.5203	584.89
Eye 7-2	5	0.0370	8.6379	3.1669	0.4055	4.5732	0.1638	186.89
Eye 8-1	5	0.0469	10.2505	3.3607	0.3425	4.4920	0.2858	410.11
Eye 8-2	5	0.0427	10.2505	3.3607	0.3370	4.5347	0.2714	452.47
Eye 8-3	5	0.0423	10.2505	3.3607	0.4165	4.9783	0.1932	191.62
Eye 8-4	5	0.0429	10.2505	3.3607	0.3135	5.4068	0.3756	687.52
Eye 9-1	5	0.0361	9.1110	3.2283	0.3465	3.7063	0.1774	327.54
Eye 9-2	5	0.0350	9.1110	3.2283	0.2645	3.6963	0.2943	617.81
Eye 10-2	5	0.0408	9.6223	3.2903	0.2870	3.9674	0.3128	587.60
Eye 10-3	5	0.0398	9.6223	3.2903	0.3000	4.1832	0.2944	637.34
Eye 10-4	5	0.0459	9.6223	3.2903	0.3540	4.4653	0.2603	386.18
